# Rapid Identification and Systematic Mechanism of Flavonoids from *Potentilla freyniana* Bornm. Based on UHPLC-Q-Exactive Orbitrap Mass Spectrometry and Network Pharmacology

**DOI:** 10.1155/2021/6619959

**Published:** 2021-01-27

**Authors:** Wei Cai, Kailin Li, Shihan Qin, Pei Xiong, Jie Peng, Silin Shi, Zaiqi Zhang

**Affiliations:** ^1^Hunan Provincial Key Laboratory of Dong Medicine, Hunan University of Medicine, Huaihua 41800, China; ^2^School of Pharmaceutical Sciences, Hunan University of Medicine, Huaihua 41800, China; ^3^School of Pharmacy, Weifang Medical University, Weifang 261053, China

## Abstract

*Potentilla freyniana* Bornm. (P. freyniana), belonging to the family Rosaceae, has been used as a folk medicine in China. However, as we know, the constituents and the systematic elucidation of the mechanism were not fully investigated. Therefore, it is necessary to develop a rapid method using LC-MS and network pharmacology for the detection and identification of constituents and the systematic mechanism of *P. freyniana*. Firstly, the flavonoids were detected and identified based on ultra-high-performance liquid chromatography coupled with Quadrupole-Exactive Focus Orbitrap MS (UHPLC-Q-Exactive Orbitrap MS). After that, the potential targets of those constituents were obtained by database mining. Then, the core targets were predicted by protein-protein interaction network and network analysis. Finally, Gene Ontology (GO) and Kyoto Encyclopedia of Genes and Genomes (KEGG) pathway enrichment analysis were carried out via DAVID. This finding revealed that *P. freyniana* possessed 43 flavonoids (40 of them were first reported) with 23 core target genes, which are associated with PI3K-Akt, MAPK, TNF signaling pathway, and pathway in cancer. This study demonstrated the multicompound, multitarget, and multimechanism of *P. freyniana*, which are very beneficial to develop the further study and utilization of this plant including the material basis and quality control research.

## 1. Introduction

*Potentilla freyniana* Bornm. (*P. freyniana*), a genus *Potentilla* of the family Rosaceae, named Difengzi, is a perennial plant with branched and tufted roots widely distributed and cultivated all-around of China, especially in Hunan, Hubei, Jiangxi. Their roots have been used as a folk medicine in clearing away heat and toxic materials for treating canker, bone *tuberculosis*, external bleeding [1–3]. Previous investigations on *P. freyniana* showed the presence of different compounds including terpenes and flavonoids [4–7] and possessed a variety of activities such as anti-inflammatory, analgesic effects [8, 9]. However, as we know, the constituents and systematic pharmacological mechanism were not fully investigated. For instance, 14 compounds, including eriodictyol, phlorizin, were separated from the roots of *P. freyniana* [7]. Therefore, it is worthwhile to establish a highly sensitive method for characterizing their chemical constituents and elucidating systematic pharmacological mechanism of *P. freyniana*.

The complexity of chemicals in Traditional Chinese Medicine (TCM) including *P. freyniana* has presented a significant challenge in the rapid identification and characterization of components. During the past decades, HPLC-MS, as a new technique has been used to profile and identify the chemical in TCM due to its validity, sensitivity, and specialness [10, 11]. Especially, UHPLC-HRMS such as UHPLC-Q-Exactive Orbitrap MS, UHPLC-Q-TOF MS, and UHPLC–LTQ-Orbitrap-MS, affording a higher and faster separation and higher resolution of mass, was a much more powerful equipment in the identification of TCM compared to traditional HPLC-MS [12–14].

Network pharmacology is an impressive methodology for investigating the systematic pharmacological mechanism through the constructing and analyzing biological networks such as protein-protein interaction, chemical-target-pathway network, which could provide direction for the further discovery of new drug without enormous time, money, and effort [15–17].

Therefore, this current study was designed to develop a fast and effective method for the chemical characterization and systematic pharmacological mechanism of *P. freyniana* using UHPLC-Q-Exactive Orbitrap MS and network pharmacology. *P. freyniana* possessed 43 flavonoids (40 of them was first reported) with 23 core target genes, which are associated with PI3K-Akt, MAPK, TNF signaling pathway, and pathway in cancer. This study demonstrated the multicompound, multitarget, and multimechanism of *P. freyniana*, which are very beneficial for the further study and utilization of this plant including the material basis and quality control research.

## 2. Materials and Methods

### 2.1. Chemicals and Materials

The chemical reference standards isoquercitrin, luteolin, naringenin, and kaempferol were provided by Chengdu Herbpurify biotechnology CO., LTD (Chengdu, China); Phlorizin, Phloretin, and Trilobatin were purchased from Sichuan Wei Keqi Biotechnology Co., Ltd (Sichuan, China); eriodictyol and hyperoside were provided by Chengdu Push biotechnology CO., LTD (Chengdu, China); quercetin and apigenin were provided by Chengdu Alfa biotechnology CO., LTD (Chengdu, China); baicalein and wogonin were obtained from Chengdu Desite biotechnology CO., LTD (Chengdu, China). The purities of all chemical reference standards were above 98% by HPLC-DAD.

Acetonitrile and methanol of chromatography grade were provided by MERCK (Darmstadt, Germany); The ultrapure water was produced by a milli-Q water purification system (Millipore, Milford, MA, United States); formic acid of LC-MS grade and all other reagents of analytical grade were purchased from Aladdin Industrial Corporation.

*P. freyniana* was collected from Tong-Dao country of Huaihua (109.86 longitude, 26.03 latitude), Hunan province and were identified by Professor Wei Cai (Hunan Provincial Key Laboratory of Dong Medicine, Hunan University of Medicine). The voucher specimen was deposited at School of Pharmaceutical Sciences, Hunan University of Medicine.

### 2.2. Sample Preparation

A total of 10 g powdered root of *P. freyniana* was ultrasonically extracted with 50 mL of 70% aqueous methanol for 1 h, and then the extracted solution was filtered for further UHPLC Q-Exactive Focus Orbitrap MS analysis.

The reference standards including hyperoside, isoquercitrin, phlorizin, eriodictyol, trilobatin, quercetin, luteolin, naringenin, apigenin, phloretin, kaempferol, baicalein, and wogonin were weighed and dissolved in methanol to obtain the reference solution with the final concentrations of 10.2, 9.8, 10.0, 9.8, 9.9, 10.5, 10.2, 10.8, 9.3, 10.1, 10.6, 9, 4, and 10.5 ug/mL, respectively, and then these solutions were stored in −4°C before analysis.

### 2.3. Instruments and Conditions

Chromatography analysis was performed on an Ultimate 3000 focused system (Dionex, Sunnyvale, CA, USA) consisting of an online vacuum degasser, a binary pump, and an autosampler. The sample separation was carried out on the Hypersil GOLD C18 column (100 × 2.1 mm, 1.9 *μ*m) at 40°C. The mobile phase consisted of acetonitrile as solvent A and water with 0.1% formic acid as solvent B using the following gradient elution of 5–15 % A at 0–2 min; isocratic 15 % A at 2–5 min; 15–18 % A at 5–10 min; 18–50 % A at 10–15 min; 50–100 % A at 15–16 min; 100–5 % A at 16–17 min; isocratic 5 % A at 17–20 min. The flow rate was 0.3 mL/min.

All LC-MS^n^ analyses were performed on the Q-Exactive Focus Orbitrap MS connected to the UHPLC system via a heated electrospray ionization source (Thermo Electron, Bremen, Germany). The optimized tune operating parameters in negative ion mode were listed as follows: sheath gas and auxiliary gas flow rate of 30 and 10 arbitrary, respectively; the capillary and auxiliary gas heater temperatures of 320°C and 350°C, respectively; spray voltage of 3.0 kV; RF lens of 50; High-resolution MS analysis was performed at full scan MS^1^ with the mass range of m/z 100–1000 at a resolution of 35000 and targeted MS^2^ at a resolution of 17500 triggered by parallel reaction monitoring mode; nitrogen was set as sheath, auxiliary, and collision gas; the isolation widow was 2 amu, and the normalized collision energy (NCE) was 30%.

### 2.4. Data Processing and Analysis

All high-resolution MS data were acquired and processed using the Xcalibur version (2.0 software, Thermo Fisher Scientific, San Jose, CA, USA). The compounds were detected by the Compound Discover version 3 using the metabolism workflow templates by the expected compounds predicted method [18]. The detailed parameters of the workflow template were set as follows: the minimum peak intensity was set as 10000; the maximum element counts were C30 H60 O20; the mass tolerance of MS and MS^2^ was within 5 and 10 ppm, respectively; baicalein and phloretin were set as the carbon skeleton; reduction, oxidation was set as Phase I transformation; glucoside conjugation, glucuronide conjugation, pentoside conjugation, methylation was set as Phase II transformation.

### 2.5. Target Identification of Flavonoids

TCMSP database is a free and online database for potential target identification of small molecules, especially TCM. The target genes were converted to the official gene symbol by STRING (https://string-db.org) or Uniport (https://www.uniprot.org).

### 2.6. Protein-Protein Interaction Network

STRING was a free tool, which can construct the PPI network by uploading the potential targets. The species was set as “*Homo sapiens*” with a confidence score >0.4. The network analysis was performed at Cytoscape to obtain the core targets.

### 2.7. GeneMANIA Analysis

GeneMANIA (http://genemania.org) is an online free and friendly tool for investigating gene function and gene interaction. The species was set as “*Homo sapiens*.”

### 2.8. GO and Pathway Analysis

The GO and KEGG pathway analysis was performed on the DAVID (https://david.ncifcrf.gov, Version 6.8). The specific species in the list and background was set as “*Homo sapiens*.” The entire compounds, targets, and pathway network were visualized by Cytoscape.

## 3. Results and Discussion

### 3.1. Analytical Strategy

In order to identify flavonoids fully, an analytical strategy based on UHPLC Q-Exactive Focus Orbitrap MS was established in this study. First, the sample was prepared and injected into the UHPLC Q-Exactive Focus Orbitrap MS to gain the full scan high-resolution MS data. Then, those data were processed using Compound Discover software with metabolism workflow to predict and detect the molecule of flavonoids. Third, the MS^2^ of the predicted molecule were acquired using UHPLC Q-Exactive Focus Orbitrap MS by parallel reaction monitoring mode. Finally, the compounds were identified based on the full scan MS, MS^2^ data, retention time, and bibliography.

### 3.2. Identification of Flavonoids

**Figure 1:**
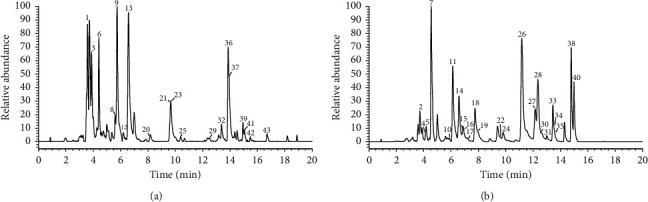
The high-resolution extracted ion chromatogram (HREIC) in 5 ppm for the multiple compounds in *Potentilla freyniana* Bornm. (a) m/z 269.0455, 283.0612, 285.0405, 301.0354, 303.0510, 431.0984, 465.1038, 479.0831, 565.1563, 567.1719, 593.1512, 625.1408; (b) m/z 271.0611, 273.0768, 287.0561, 433.1140, 435.1297, 449.1089, 463.0882, 465.1038, 477.0674, 597.1825.

The total content of flavonoids was measured by NaNO_2_-Al(NO_3_)_3_-NaOH spectrophotometric colorimetry [19]. The calibration curve obtained by the rutin standard of absorbance concentrations(mg/mL) using five dilutions was *y* = 7.15*x* − 0.001, with the corresponding determination coefficient at 0.9999. Finally, the content of flavonoids is 32.17 ± 0.26%. A total of 43 constituents were unanimously and tentatively characterized based on UHPLC Q-Exactive Focus Orbitrap MS combined with the expected compounds predicted method. 40 excluded eriodictyol, phloretin, and hyperoside were reported from *P. freyniana* for the first time. The detailed information of those compounds is listed in Table 1. The high-resolution extracted ion chromatography is shown in Figure 1.

Peaks 15, 17, 27, 33, 35–43 were unanimously identified as hyperoside, isoquercitrin, phlorizin, eriodictyol, trilobatin, quercetin, luteolin, naringenin, apigenin, phloretin, kaempferol, baicalein, and wogonin, respectively, by comparing the retention time, high-resolution mass measurement, and MS^2^ spectrum with those reference standards.

Peak 13 was eluted at 6.57 min and possessed the deprotonated ion [M−H]^−^ at m/z 303.0507 (−0.99 ppm, C_15_H_11_O_7_). The fragment ions at m/z 125.0234 (−8.14 ppm, C_6_H_5_O_3_) and 285.0407 (0.84 ppm, C_15_H_9_O_6_) were detected in the MS^2^ spectrum, which is consistent with the MS data of taxifolin in bibliography [20]. Thus, peak 13 was tentatively identified as taxifolin. Peaks 1–3, and 8 possessed the deprotonated ion [M−H]^−^ at m/z 465.1039 (0.21 ppm, C_21_H_21_O_12_), m/z 465.1033 (−1.08 ppm, C_21_H_21_O_12_), m/z 465.1042 (0.86 ppm, C_21_H_21_O_12_), and m/z 465.1042 (0.86 ppm, C_21_H_21_O_12_), respectively, 162 Da(C_6_H_10_O_5_, glucose moiety) more than that of taxifolin (peak 13). The fragmentation ions at m/z 285.041(C_15_H_9_O_6_), 125.023(C_6_H_5_O_3_), 303.051(C_15_H_11_O_7_) in the MS^2^ spectrum were matched to those attributed to taxifolin. Therefore, Peaks 1–3, and 8 were tentatively characterized as taxifolin-glucoside.

Peaks 4 and 26 were eluted at 4.13 and 11.18 min, respectively. All of them showed the same deprotonated ion [M−H]^−^ at m/z 463.088 (C_21_H_19_O_12_), 176.032 Da(C_6_H_8_O_6_, glucuronide moiety) more than that of eriodictyol, suggesting they are eriodictyol-glucuronide, which were further identified by the presence of fragmentation ion at m/z 287.056 (C_15_H_11_O_6_). In a similar way, peaks 9, 28, and 30 were tentatively identified as taxifolin-glucuronide, quercetin-glucuronide, and quercetin-glucuronide, respectively.

Peaks 5, 7, 10, 16, 19, 22, and 34 were eluted at 4.19, 4.56, 5.88, 6.90, 7.99, 9.61, and 13.57 min, with the same deprotonated ion [M−H]− at m/z 449.109 (C_21_H_21_O_11_). Peaks 19 and 34 possessed the fragment ions at m/z 167.034 (C_8_H_7_O_4_) and m/z 123.044 (C_7_H_7_O_2_), which are the diagnosis fragmentation ions of phloretin, suggesting they were phloretin derivatives. Thus, Peaks 19 and 34 were tentatively inferred as phloretin-glucuronide. Peaks 5, 7, 10, 16, and 22 yielded the same fragmentation ion at m/z 287.056 (C_15_H_11_O_6_), suggesting they were eriodictyol derivatives. The ion at m/z 287.056 was yielded by the neutral loss of 162.053 (C_6_H_10_O_5_, glucose moiety), suggesting the presence of glucose moiety. Therefore, they were tentatively characterized as eriodictyol-glucoside.

Peak 6 with the deprotonated ion [M−H]− at m/z 593.1535 (3.88 ppm, C_27_H_29_O_15_) was eluted at 4.41 min. It yielded fragment ions at m/z 353.0667 (0.07 ppm, C_19_H_13_O_7_), 383.0774 (0.42 ppm, C_20_H_15_O_8_), 473.1092(0.56 ppm, C_23_H_21_O_11_), and 413.0874 (−0.98 ppm, C_21_H_17_O_9_), resulting from the loss of C_4_H_8_O_4_ + C_4_H_8_O_4_, C_4_H_8_O_4_ + C_3_H_6_O_3_, C_4_H_8_O_4_, and C_3_H_6_O_3_ + C_3_H_6_O_3_, respectively, suggesting the presence of two carbon-glucoside. According to the published paper [21, 22], peak 6 was tentatively identified as Vicenin II. In a similar way, peak 18 was tentatively identified as Phloretin-C-diglucoside.

Peaks 11, 14, 24, and 31 generated the same quasimolecular ion [M−H]− at m/z 433.114 (C_21_H_21_O_10_), 162 Da(C_6_H_10_O_5_, glucose moiety) more than that of naringenin (peak 38), suggesting they were naringenin-glucoside, which were further confirmed by the presence of m/z 271.061 and 151.003 in MS^2^ spectrum.

Peak 12 eluted at 6.22 min and showed a pseudomolecular ion at m/z 625.1408 (0.00 ppm, C_27_H_29_O_17_), 176.032 Da(C_6_H_8_O_6_, glucuronide moiety) more than that of eriodictyol-glucoside, suggesting it is eriodictyol-glucoside-glucuronide, which was confirmed by the presence of the base peak at m/z 287.0558 (eriodictyol).

Peaks 20, 21, 25, and 29 presented the same deprotonated ion [M−H]− at m/z 431.099 (C_21_H_19_O_10_) and generated the same fragment ions at m/z 269.044 (C_15_H_9_O_5_) by loss of the glucose moiety (C_6_H_10_O_5_), which suggested the presence of glucose moiety. The base peak at m/z 268.037 [Y_0_–H] ions in the MS^2^ spectrum of peaks 25 and 29 was a characteristic of apigenin aglycone. According to the published paper [23, 24], they were tentatively inferred as apigenin-7-glucoside and apigenin-4′-glucoside, respectively. Meantime, peaks 20 and 21 were tentatively characterized as baicalein-glucoside.

Peak 32 was detected at 13.39 min. It presented a pseudomolecular ion at m/z 567.1730 (1.94 ppm, C_26_H_32_O_14_) and exhibited the MS^2^ fragmentation ions at m/z 273.0771 (1.10 ppm, C_15_H_13_O_5_), resulting from the loss of glucose moiety and pentoside moiety (294.096). Thus, peak 32 was tentatively characterized as phloretin-pentoside-glucoside. In a similar way, peak 23 was tentatively identified as naringenin-pentoside-glucoside.

### 3.3. Target Identification of Flavonoids

212 putative targets of flavonoids were obtained from the TCMSP database. A visual compounds-targets network with 224 nodes and 440 edges was built by Cytoscape Version 3.7.2 (Figure S1). Compounds quercetin, apigenin, kaempferol, luteolin, and wogonin are the top 5 compounds with a maximum degree and betweenness in the compound-targets network. The detailed information of putative targets linked to compounds was provided in Supplementary Table S1.

### 3.4. Protein-Protein Interaction Network

In order to find the key targets of flavonoids, a total of 212 putative targets were imported into the STRING to obtain the protein-protein interaction (PPI) data. The PPI network with 206 nodes and 3980 edges was established by Cytoscape (Figure S2). A total of 23 targets, including AKT1, INS, TP53, IL6, HSP90AA1, EGFR, VEGFA, JUN, EGF, CASP3, MAPK1, ESR1, ERBB2, PTGS2, MYC, MAPK8, MMP9, FN1, FOS, PPARG, CXCL8, CYCS, and CCND1, were selected as the core targets for GO and KEGG pathway analysis by setting the parameters as follows: the degree ≥50; betweenness centrality ≥0.01; closeness centrality ≥0.6.

### 3.5. GeneMANIA Analysis

**Figure 2:**
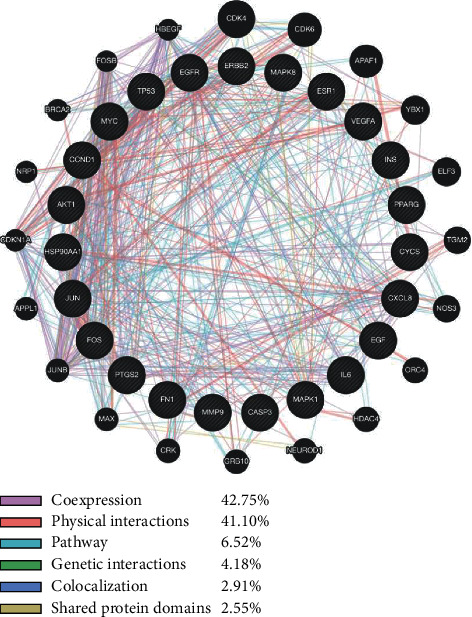
Protein network of core target genes by GeneMANIA. Black nodes represent target proteins, and connecting colors indicate different correlations.

Among the 23 key target genes and their interacting genes, it was found that 42.75 % had coexpression characteristics, 41.10 % displayed physical interactions characteristic. Other characteristics, including pathway, genetic interactions, colocalization, and shared protein domains, are displayed in Figure 2.

### 3.6. GO and Pathway Analysis

**Figure 3:**
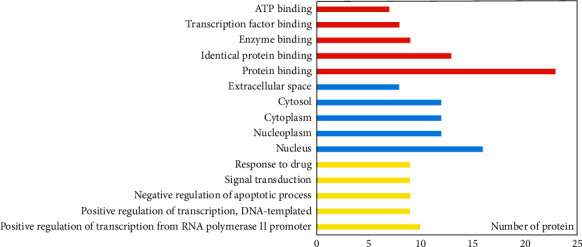
GO analysis by DAVID: red represents the biological process, yellow represents the cellular component, green represents the molecular function.

**Figure 4:**
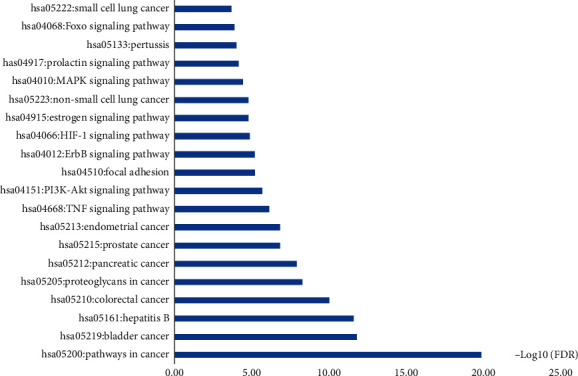
KEGG pathway analysis.

In order to further study the 23 core target genes, GO and KEGG pathway analysis were performed by DAVID. GO term enrichment analysis results were divided into the biological process (BP, 23/23), cell compound (CC, 23/23), and molecular function (MF, 23/23). A total of 158 BP, 16 CC, and 33 MF has a *p*-value less than 0.05 (Table S2). In GO term enrichment analysis, the BP might be related to positive regulation of transcription from RNA polymerase II promoter (10/23), response to drug (9/23), negative regulation of apoptotic process (9/23), positive regulation of transcription, DNA-templated (9/23), signal transduction (9/23), positive regulation of gene expression (8/23), and positive regulation of cell proliferation (8/23), and so on. The top 4 of CC are nucleus (16/23), nucleoplasm (12/23), cytosol (12/23), and cytoplasm (12/23). The MF are protein binding on 100%, identical protein binding on 56.5%, enzyme binding on 39.1%, and transcription factor binding on 34.8%. The top 10 enriched terms in BP, CC, and MF are displayed in Figure 3. In addition, 83 KEGG pathways (Table S3) were enriched as *p*-value less than 0.05. The result showed that the pathway was mainly related to the signaling pathway including PI3K-Akt (12/23), MAPK (10/23), TNF (9/23), ErbB (8/23), HIF-1 (8/23), Estrogen (8/23), FoxO (8/23), and cancer in the pathway. The top 20 KEGG pathways are shown in Figure 4.

### 3.7. Network Analysis

**Figure 5:**
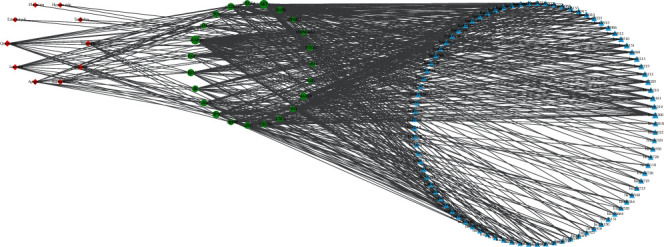
Chemical-target genes-pathway network: red represents chemical; blue represents target genes; green represents pathway.

Based on the target and KEGG pathway analysis, the entire compounds, targets, and pathway network were established by Cytoscape. The network with 122 nodes and 595 edges is shown in Figure 5. The red diamond, green ellipse, and blue triangle represent compounds, genes, and pathways, respectively.

## 4. Conclusion

In the present investigation, this finding revealed that *P. freyniana* possessed 43 flavonoids (40 of them was first reported) with 23 core target genes, which were associated with PI3K-Akt, MAPK, TNF signaling pathway, and pathway in cancer. This study demonstrated the multicompound, multitarget, and multimechanism of *P. freyniana*, which are very beneficial for the further study and utilization of this plant including the material basis and quality control research.

## Figures and Tables

**Table 1:** The retention time and mass spectrometric data of flavone in *Potentilla freyniana* Bornm.

Peak	*t*_*R*_	Theoretical mass, m/z	Experimental mass, m/z	Error (ppm)	Formula	MS/MS fragment	Identification
1	3.59	465.1038	465.1039	0.21	C_21_H_22_O_12_	MS^2^[465]: 285.0406(100), 125.0233(72), 275.0563(45), 177.0185(29), 303.0512(20), 151.0032(17)	Taxifolin-glucoside
2	3.72	465.1038	465.1033	−1.08	C_21_H_22_O_12_	MS^2^[465]: 285.0405(100), 125.0233(38), 273.0043(28), 177.0185(19), 303.0511(15)	Taxifolin-glucoside
3	3.88	465.1038	465.1042	0.86	C_21_H_22_O_12_	MS^2^[465]: 285.0405(100), 125.0233(36), 177.0184(18), 275.0566(12), 303.0512(10)	Taxifolin-glucoside
4	4.13	463.0882	463.0881	−0.22	C_21_H_20_O_12_	MS^2^[463]: 287.0562(100), 259.0612(78), 125.0232(32)	Eriodictyol-glucuronide
5	4.19	449.1089	449.1093	0.89	C_21_H_22_O_11_	MS^2^[449]: 259.0611(100), 287.0561(33), 178.9979(15), 125.0231(13)	Eriodictyol-glucoside
6	4.41	593.1512	593.1535	3.88	C_27_H_30_O_15_	MS^2^[593]: 353.0667(100), 383.0774(54), 473.1092(35), 125.0234(25), 413.0874(6)	Vicenin II
7	4.56	449.1089	449.1092	0.67	C_21_H_22_O_11_	MS^2^[449]: 259.0612(100), 269.0455(76), 287.0563(44), 125.0233(38), 178.9977(22)	Eriodictyol-glucoside
8	5.61	465.1038	465.1042	0.86	C_21_H_22_O_12_	MS^2^[465]: 125.0233(100), 285.0405(62), 259.0610(48), 275.0566(32), 303.0512(18)	Taxifolin-glucoside
9	5.75	479.0831	479.0834	0.63	C_21_H_20_O_13_	MS^2^[479]: 285.0405(100), 125.0233(41), 303.0512(18), 177.0186(17), 169.0133(15), 259.0613(14)	Taxifolin-glucuronide
10	5.88	449.1089	449.1092	0.67	C_21_H_22_O_11_	MS^2^[449]: 269.0456(100), 151.0026(69), 178.9979(39), 125.0231(13), 259.0612(12), 287.0562(10)	Eriodictyol-glucoside
11	6.12	433.1140	433.1142	0.46	C_21_H_22_O_10_	MS^2^[433]: 271.0614(100), 151.0027(73). 119.0491(18), 125.0229(8)	Naringenin-glucoside
12	6.22	625.1408	625.1408	0.00	C_27_H_30_O_17_	MS^2^[625]: 287.0558(100), 113.0231(40), 151.0030(38)	Eriodictyol-glucoside-glucuronide
13	6.57	303.0510	303.0507	−0.99	C_15_H_12_O_7_	MS^2^[303]: 125.0234(100), 285.0407(38)	**Taxifolin**
14	6.59	433.1140	433.1141	0.23	C_21_H_22_O_10_	MS^2^[433]: 271.0613(100), 151.0027(72), 119.0491(16)	Naringenin-glucoside
15 ^*∗*^	6.85	463.0882	463.0886	0.86	C_21_H_20_O_12_	MS2[463]: 300.0272(100), 301.0350(70)	Hyperoside
16	6.90	449.1089	449.1094	1.11	C_21_H_22_O_11_	MS^2^[449]:151.0028(100), 287.0562(73), 135.0442(32)	Eriodictyol-glucoside
17 ^*∗*^	7.18	463.0882	463.0886	0.86	C_21_H_20_O_12_	MS2[463]: 300.0279(100), 301.0358(50), 151.0025(8), 178.9976(7)	Isoquercitrin
18	7.75	597.1825	597.1832	1.17	C_27_H_34_O_15_	MS^2^[597]: 357.0983(100), 387.1087(85), 315.0882(22), 417.1165(21), 358.1008(15)	Phloretin-C- diglucoside
19	7.99	449.1089	449.1085	−0.89	C_21_H_22_O_11_	MS^2^[449]: 167.0340(69), 137.0233(50), 123.0441(12)	Phloretin-glucuronide
20	8.15	431.0984	431.0992	1.86	C_21_H_20_O_10_	MS^2^[431]: 269.0445(100)	Baicalein-glucoside
21	9.60	431.0984	431.0988	0.93	C_21_H_20_O_10_	MS^2^[431]: 269.0443(100)	Baicalein-glucoside
22	9.61	449.1089	449.1086	−0.67	C_21_H_22_O_11_	MS^2^[449]: 151.0026(100), 287.0563(65), 135.0442(22)	Eriodictyol-glucoside
23	9.68	565.1563	565.1568	0.88	C_26_H_30_O_14_	MS^2^[565]: 271.0614(100), 151.0026(48)	Naringenin-pentoside-glucoside
24	9.82	433.1140	433.1142	0.46	C_21_H_22_O_10_	MS^2^[433]: 271.0615(100), 151.0028(41), 119.0491(14)	Naringenin-glucoside
25	10.38	431.0984	431.0992	1.86	C_21_H_20_O_10_	MS^2^[431]: 268.0369(100), 269.0444(47)	Apigenin-7-glucoside
26	11.18	463.0882	463.0886	0.86	C_21_H_20_O_12_	MS^2^[463]: 151.0027(100), 113.0231(66), 287.0562(52), 161.0234(40), 337.0569(39), 135.0439(23)	Eriodictyol-glucuronide
27 ^*∗*^	12.16	435.1297	435.1300	0.69	C_21_H_24_O_10_	MS^2^[435]: 273.0769(100), 167.0340(69), 125.00232(9), 179.0341(6)	Phlorizin
28	12.36	477.0674	477.0678	0.84	C_21_H_18_O_13_	MS^2^[477]: 301.0353(100), 151.0029(11), 178.9983(9)	Quercetin-glucuronide
29	12.51	431.0984	431.0986	0.46	C_21_H_20_O_10_	MS^2^[431]: 268.0371(100), 269.0441(18), 239.0337(9)	Apigenin-4′-glucoside
30	12.62	477.0674	477.0678	0.84	C_21_H_18_O_13_	MS^2^[477]: 301.0354(100), 178.9983(12), 151.0029(8)	Quercetin-glucuronide
31	13.00	433.1140	433.1144	0.92	C_21_H_22_O_10_	MS^2^[433]: 271.0612(100), 151.0027(46)	Naringenin-glucoside
32	13.39	567.1719	567.1730	1.94	C_26_H_32_O_14_	MS^2^[567]: 273.0771(100), 167.0340(42), 125.0020(22)	Phloretin-pentoside-glucoside
33 ^*∗*^	13.45	287.0561	287.0564	1.05	C_15_H_12_O_6_	MS2[287]: 151.0027(100), 135.0441(69), 107.0125(10)	Eriodictyol
34	13.57	449.1089	449.1085	−0.89	C_21_H_22_O_11_	MS^2^[449]: 137.0233(100), 167.0340(89), 123.0440(8)	Phloretin-glucuronide
35 ^*∗*^	13.67	435.1297	435.1303	1.38	C_21_H_24_O_10_	MS^2^[435]: 273.0767(100), 167.0339(52)	Trilobatin
36 ^*∗*^	13.86	301.0354	301.0356	0.66	C_15_H_10_O_7_	MS^2^[301]: 151.0027(100), 178.9978(66), 121.0284(21), 107.0125(8)	Quercetin
37 ^*∗*^	13.95	285.0405	285.0407	0.70	C_15_H_10_O_6_	MS^2^[285]: 285.0404(100), 151.0033(12), 133.0290(11), 175.0395(8)	Luteolin
38 ^*∗*^	14.79	271.0611	271.0614	1.11	C_15_H_12_O_5_	MS^2^[271]: 151.0027(100), 119.0499(36), 177.0184(14), 93.0333(14), 107.0126(9)	Naringenin
39 ^*∗*^	14.95	269.0455	269.0454	−0.37	C_15_H_10_O_5_	MS^2^[269]: 269.0455(100), 117.0344(8), 149.0239(7), 151.0035(7)	Apigenin
40 ^*∗*^	14.99	273.0768	273.0771	1.10	C_15_H_14_O_5_	MS^2^[273]: 167.0341(100), 123.0438(18), 119.0491(12),125.0231(8)	Phloretin
41 ^*∗*^	15.07	285.0405	285.0405	0.00	C_15_H_10_O_6_	MS^2^[285]: 285.0403(100), 151.0030(5)	Kaempferol
42 ^*∗*^	15.49	269.0455	269.0453	−0.74	C_15_H_10_O_5_	MS^2^[269]: 269.0453(100), 241.0504(9), 251.0348(8), 223.0399(7)	Baicalein
43 ^*∗*^	16.71	283.0612	283.0613	0.35	C_16_H_12_O_5_	MS^2^[283]: 268.0376(100), 163.0035(7)	Wogonin

^*∗*^Identified by comparing with reference standards.

## Data Availability

The data used to support the finding of this study are available from the corresponding author upon request.

## Supplementary Materials

Supplementary Materials Figure S1: compounds-target genes. Figure S2: PPI network. Table S1: compounds-target genes. Table S2: GO analysis. Table S3: KEGG pathway.
